# The combination of Shenhuang plaster and paclitaxel inhibits lung metastasis in breast cancer via modulation of the tumor microenvironment

**DOI:** 10.3389/fonc.2025.1531493

**Published:** 2025-02-28

**Authors:** Shiqi Chen, Maryam Mohammed Abbas Karekad, Ting Liu, Bin Ding, Rongyun Wang, Qiuhua Sun, Xiaohong Xu, Yanan Shi

**Affiliations:** ^1^ The First Clinical Medical College of Zhejiang Chinese Medical University, Hangzhou, China; ^2^ The International Education College, Zhejiang Chinese Medical University, Hangzhou, China; ^3^ The College of Nursing, Zhejiang Chinese Medical University, Hangzhou, China; ^4^ College of Life Science, Zhejiang Chinese Medical University, Hangzhou, China; ^5^ The First Affiliated Hospital of Zhejiang Chinese Medical University (Zhejiang Provincial Hospital of Traditional Chinese Medicine), Hangzhou, China; ^6^ Bozhou District Hospital of Traditional Chinese Medicine, Zunyi, China

**Keywords:** Shenhuang plaster, breast cancer, lung metastasis, tumor microenvironment, Paclitaxel

## Abstract

**Background:**

Paclitaxel (PTX) is a chemotherapeutic agent that is frequently used for breast cancer treatment, but it has been associated with promoting distant metastases, including to the lungs, liver, and bones. Shenhuang plaster (SHP), a traditional Chinese medicine, has shown potential for modulating the tumor microenvironment (TME). This study investigates whether a combination of SHP and PTX can enhance the anti-tumor efficacy of PTX and mitigate its pro-metastatic effects in a 4T1 breast cancer mouse model.

**Methods:**

Female Balb/c mice were injected with 4T1 breast cancer cells and then divided into four treatment groups: control, PTX, SHP, and PTX+SHP. The combination of SHP and PTX was evaluated using bioluminescence imaging (BLI), histological analysis, and hematoxylin and eosin (HE) staining to assess lung metastasis. Flow cytometry was employed to analyze immune cell populations, including tumor-associated macrophages (TAMs), myeloid-derived suppressor cells (MDSCs), regulatory T cells (Tregs), and cytotoxic T cells (CD8+ and CD4+).

**Results:**

SHP alone did not significantly inhibit lung metastasis but the combination of PTX and SHP led to a marked reduction in lung lesions, as confirmed by BLI and histological analysis. SHP improved the overall health of PTX-treated mice, reducing their body weight loss and mortality. Flow cytometry revealed that the combination therapy reduced the infiltration of M2 macrophages, MDSCs, and Tregs, while increasing the proportion of antitumor M1 macrophages, cytotoxic CD8+ T cells, and helper CD4+ T cells.

**Conclusions:**

The combination of PTX and SHP has a synergistic effect, reducing lung metastasis and modulating immune cell populations within the TME. These results suggest that integrating traditional Chinese medicine with standard chemotherapy can enhance therapeutic efficacy and reduce adverse effects.

## 1 Introduction

Breast, lung, and colorectal cancers are the three most frequently diagnosed cancers among females with increasing incidences and deaths globally (1). In 2020, 30% of all new cancer cases and 15% of all cancer deaths were attributed to breast cancer among American women (2). Clinically, distant metastases (DM) remain the main cause of death. Although 90% of patients with DM show a poor prognosis and even death (3, 4), no effective treatment is available for metastatic breast cancer, including lung and bone metastases (5). Lung metastases are a major cause of mortality in breast cancer patients, often occurring in advanced stages of the disease. Paclitaxel (PTX), commonly known as Taxol, is a microtubule-stabilizing chemotherapeutic agent that is frequently used to treat breast cancer and other solid tumors (6). PTX works by disrupting the mitotic spindle, leading to cell cycle arrest and apoptosis in rapidly dividing cancer cells. Despite its efficacy, prolonged treatment with PTX has been associated with promoting epithelial-mesenchymal transition (EMT), which contributes to the enhanced potential for metastasis, including lung metastasis (7–9). This phenomenon limits the effectiveness of PTX as a long-term solution for controlling metastasis in breast cancer patients.

The evolution of breast cancer is closely associated with the surrounding tumor microenvironment (TME). Tumor-infiltrating immune cells are key components of the TME and can have both anti- and pro- tumorigenic effects (10, 11). Inflammation and the infiltration of innate immune cells, including macrophages and neutrophils, are required to fight infection. In the case of cancer, however, cancer cells develop several mechanisms to evade immune destruction, including the secretion of cytokines that directly suppress cytotoxic CD8^+^ T cells and recruit regulatory T cells (Tregs) and myeloid-derived suppressor cells (MDSCs) (12, 13). Moreover, it has been demonstrated that the accumulation of tumor-associated macrophages (TAMs) is closely associated with a poor prognosis in breast cancer patients (10). Breast tumors regulate transitions in mammary gland homeostasis, and the same process is also observed in metastases (14). In recent years, the TME has been linked to a regulatory role in all stages of breast cancer development and is associated with the prognosis, nodal metastasis, and DM in various types of cancers; thus, the TME has been proposed as a potential target for cancer therapy (13).

Shenhuang plaster (SHP) is a ‘Qi’-promoting herbal formula consisting of Renshen (Ginseng Radix Et Rhizoma), raw Dahuang (Rhei Radix Et Rhizoma), Danshen (Salviae Miltiorrhizae Radix Et Rhizoma), Zhishi (Aurantii Fructus Immaturus), Houpo (Magnoliae Officinalis Cortex), Dingxiang (Caryophylli Flos), and Wuzhuyu (Euodiae Fructus). SHP has been applied clinically on Shenque (CV8), improving chemotherapeutic constipation in breast cancer patients for many years (15). In our previous animal studies, we elucidated the pharmacological mechanism of SHP treatment, demonstrating that SHP improves the expression of gastrointestinal motility hormones, such as ghrelin, motilin, and vasoactive intestinal peptide (VIP), as well as inhibiting the expression and secretion of some inflammatory cytokines (16). In short, SHP has been shown possess anti-inflammatory, immune-modulating, and detoxifying properties, indicating that it influences the TME by alleviating inflammation, regulating immune responses, and potentially inhibiting tumor growth. However, the specific mechanism(s) by which SHP affects cancer metastasis, particularly in combination with standard chemotherapeutics like PTX, are not fully understood. Therefore, the present study aimed to determine whether SHP can enhance the anti-cancer effects of PTX while mitigating its metastasis-promoting side effects.

## 2 Materials and methods

### 2.1 Chemicals and reagents

The SHP formula was provided by Prof. Xu Zhiying from Zhejiang Chinese Medical University, Zhejiang, China. SHP was prepared according to previously reported methods, and its ingredients were analyzed and identified using UPLC-MS/MS (16). PTX (No. 20053001) was obtained from Yangzijiang Pharmaceutical (Taizhou, China), and the 4T1-luc breast cancer cells were generously provided by Dr. Gao (Zhejiang Chinese Medical University, China). All antibodies used in flow cytometry analyses were obtained from BD Biosciences (Franklin Lakes, NJ, USA) and are listed in **Supplementary Table S1**. The culture media, including CTL medium, RPMI 1640 medium, DMEM, and fetal bovine serum (FBS), were procured from Gibco (Invitrogen Co., Carlsbad, CA, USA). Penicillin and streptomycin were purchased from Harbin Pharmaceutical Group Co., Ltd. (Heilongjiang, China). Red blood cell lysis buffer and MACS buffer were obtained from Sigma-Aldrich (St. Louis, MO, USA), and fluorescein substrate was obtained from Keyuandi Biotechnology Co., Ltd. (Shanghai, China). Anhydrous ethanol, eosin, and other chemical reagents were purchased from East China Pharmaceutical (Hangzhou, China).

### 2.2 Experimental instruments

The flow cytometry equipment was purchased from BD Biosciences (Franklin Lakes, NJ, USA), and 70-μm cell strainers were obtained from BD Falcon Compa Biosciences ny (Franklin Lakes, NJ, USA). The Xenogen IVIS 200 imaging system was purchased from Caliper Life Sciences (Hopkinton, MA, USA).

### 2.3 Cell line

The 4T1 breast cancer cell line was derived from spontaneous breast tumors (17). 4T1 cells were cultured in RPMI 1640 medium containing 10% FBS and antibiotics (50 IU/mL penicillin and 50 μg/mL streptomycin) at 37°C in a 100% humidified incubator. The medium was refreshed every two days until a sufficient number of cells was obtained for the experiments. Cells were collected, washed three times with PBS, and resuspended in PBS buffer (5×10^6^ cells/1 mL). The cell suspension was used for subcutaneous injection (16).

### 2.4 Animal studies

A total of 32 adult female Balb/c mice (6–8 weeks old) were purchased from the Shanghai Laboratory Animal Center (Shanghai, China). The mice were housed under specific pathogen-free (SPF) conditions at the Animal Center of Zhejiang Chinese Medicine University (Accepted Nr. 11039) for one week, with a 12-h light/dark cycle and a constant temperature of 20°C. The mice had free access to food and water throughout the study.

All mice were anesthetized with 4% isoflurane and then were injected with 100 μL of the 4T1 cell suspension (5 × 10^5^ cells) in the fourth mammary fat pad on the right side. Two weeks after the injection, tumor nodules were observed and measured. Mice with similar tumor sizes were randomly divided into four groups (8 mice per group) as follows: a Negative Control (Ctrl) Group: Application of 100 μL saline on CV8 daily, plus an intraperitoneal (i.p.) injection of 100 μL saline three times a week; a SHP Group: Application of 500 mg SHP in 100 μL saline on CV8 daily, plus an i.p. injection of 100 μL saline three times a week; a PTX Group: Application of 100 μL saline on CV8 daily, plus an i.p. injection of 18 mg/kg body weight PTX in 0.2 mL per mouse three times a week; a PTX + SHP Group: Application of 500 mg SHP in 100 μL saline on CV8 daily, plus an i.p. injection of 18 mg/kg body weight PTX in 0.2 mL per mouse three times a week.

Mice were sacrificed with CO_2_, and lung and tumor tissues were collected and weighed. The lungs were fixed in 4% formaldehyde, and the tumors were stored in CTL medium for flow cytometry analysis.

### 2.5 Bioluminescence imaging of lung metastasis

At the end of the experiment, mice were injected i.p. with fluorescein substrate (150 mg/kg) for imaging. After 15 min, the mice were anesthetized with isoflurane and placed on the Xenogen IVIS 200 imaging system, with their chest and abdomen facing the instrument lens. Images were acquired and analyzed using LT Living Image 4.3 Software.

### 2.6 Flow cytometry analysis

Tumors were maintained in 6-well culture plates containing 2 mL CTL culture medium (RPMI 1640, 1% L-glutamine, 1% nonessential amino acids, 1% sodium pyruvate, 1% penicillin/streptomycin, 10% fetal calf serum, and 0.1% β-mercaptoethanol). Each tumor was cut into small pieces and digested with 2 mg/mL collagenase D at 37°C for 1 h. Tumor cells were separated from debris using 70-μm cell strainers (Biologix 15-1070, Tisch Scientific, Cleves, OH, USA) and collected by centrifugation at 1500 rpm for 5 min. Each cell pellet was resuspended in 5 mL red blood cell lysis buffer and incubated for 30 min at 37°C. Cells were collected by centrifugation and washed twice with MACS buffer (PBS with 2% FBS and 1% penicillin/streptomycin). Finally, cells were resuspended in 3 mL fluorescence-activated cell sorting (FACS) buffer, and fluorescent antibodies were added to hybridize with specific cells for cell sorting analysis (**Supplementary Table S1**). Representative indicators of the detected phenotypes are shown in **Supplementary Table S2**. In addition, 7-AAD (PerCP-Cy5-5-A) was used to hybridize and identify non-viable cells. Generally, 1×10^5^ cells were collected from each sorting session for subsequent analyses.

### 2.7 Histological analysis of lungs

Lungs that had been fixed in formaldehyde were successively dehydrated, embedded in paraffin, and cut into 4-μm sections. The sections were heated in a 60°C oven for 2 h and then incubated in a successive gradient according to the manufacturer’s instructions as follows: xylene I for 15 min, xylene II for 15 min, anhydrous ethanol I for 10 min, and anhydrous ethanol II for 10 min. The sections were then immersed in a series of ethanol solutions (95%, 80%, and 70%) and rinsed with tap water. Finally, the sections were stained with HE solution for 5 min. The sections were then imaged and analyzed using a NanoZoomer Digital Slice Scanner (NDP).

### 2.8 Statistical analysis

Quantitative data are presented as means ± standard (SD). Differences between groups were analyzed by one-way ANOVA using SPSS software (version 19.0, SPSS). A *p* value of less than 0.05 was considered statistically significant.

## 3 Results

### 3.1 SHP alleviates PTX-induced body weight loss in the 4T1 breast cancer mouse model

**Figure 1:**
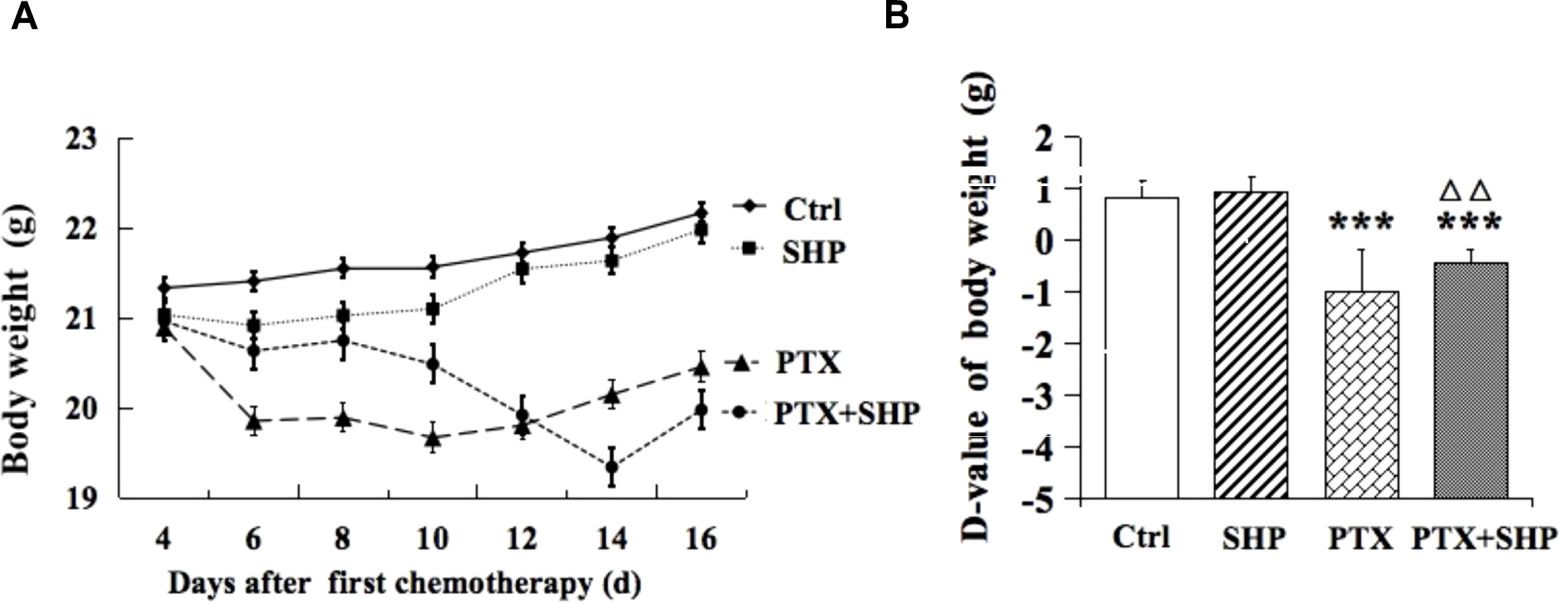
Changes in body weight of tumor bearing mice treated with SHP and/or PTX. **(A)** Body weight in each group during the chemotherapy cycle. **(B)** D-value of the body weight in the four groups. Data are presented as means ± SD (***P < 0.001 compared to the Ctrl group; △△*P* < 0.01 compared to the PTX group).

The effects of SHP on body weight were evaluated in the 4T1 breast cancer mouse model across four groups: Ctrl, SHP, PTX, and PTX+SHP. Body weight was monitored every other day during the chemotherapy cycle. Mice in the PTX group experienced significant body weight loss compared to the Ctrl group, while the PTX+SHP group showed an overall improvement in weight retention compared to the PTX group (**Figure 1A**). Although the final body weight in the PTX+SHP group appeared slightly lower than in the PTX group at the end of therapy, the trend over the treatment period indicated that SHP helped mitigate weight loss associated with PTX treatment. The D-value, calculated as the difference between the initial and final body weights, further supported these findings. The D-value was significantly higher in the PTX+SHP group compared to the PTX group, confirming reduced weight loss in the combination treatment group (**Figure 1B**). These results suggest that SHP helps maintain body weight during PTX chemotherapy, potentially improving the overall physical condition of the mice.

### 3.2 The combination of SHP and PTX effectively inhibits pulmonary metastasis in the 4T1 breast cancer mouse model

**Figure 2:**
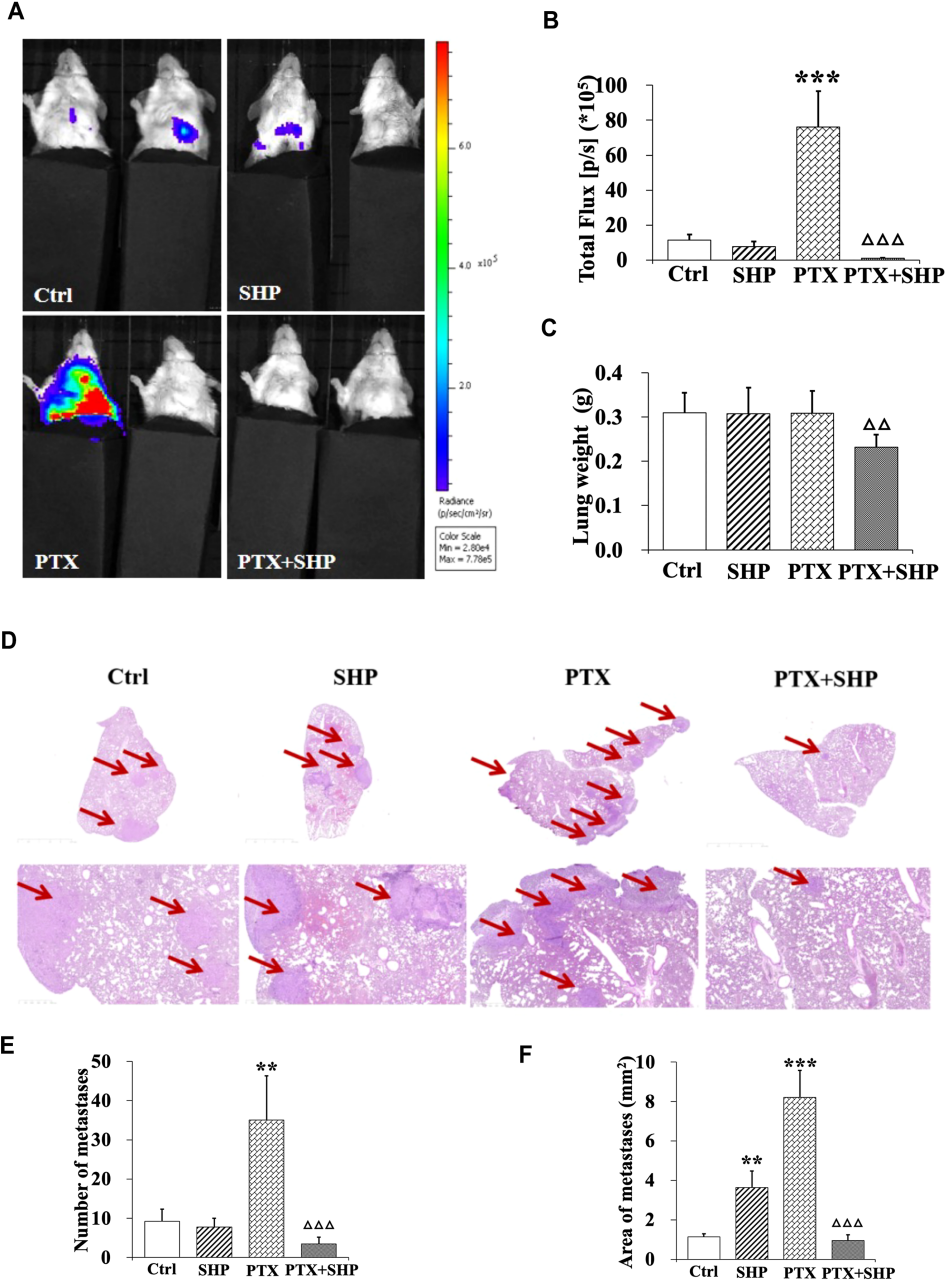
The combination of SHP and PTX reduces pulmonary metastasis in the 4T1 breast cancer mouse model. **(A)** Representative luciferin imaging of the lungs in the four groups. **(B)** Quantification of bioluminescence in the lungs in the four groups. **(C)** Lung weight in each group. **(D)** HE staining of lung tissues in the four groups (under different magnification). The red arrows indicate metastases. **(E)** Number of metastases in each group. **(F)** Area of metastases in each group. Data are presented as means ± SD (**P < 0.01, and ***P < 0.001 compared to the Ctrl group; △△P < 0.01, and △△△P < 0.001 compared to the PTX group).

Lung tissues from tumor bearing mice in the four groups (Ctrl, SHP, PTX, PTX+SHP) were analyzed to assess metastasis. First, representative bioluminescence images (BLI) showed reduced fluorescence in the PTX+SHP group compared to the PTX group, which suggests that there was less metastatic activity in the lungs (**Figure 2A**). This finding was further confirmed by quantification of bioluminescence (total flux) which demonstrated significantly reduced metastatic activity in the PTX+SHP group compared to the PTX group (**Figure 2B**). Moreover, analysis of lung weight revealed that had significantly lower lung weights compared to the PTX group (**Figure 2C**), further supporting the reduced metastatic burden in the combination treatment group. HE staining showed a marked reduction in both the number and area of lung metastases in the PTX+SHP group compared to the PTX group (**Figures 2D–F**). Red arrows in the images highlight metastatic lesions, which were notably fewer and smaller in the PTX+SHP group. These findings suggest that the combination of SHP and PTX exerts a synergistic effect in reducing lung metastasis in breast cancer, demonstrating significant improvements over PTX treatment alone.

### 3.3 SHP and PTX modulate the infiltration of inflammatory cells in solid tumors

**Figure 3:**
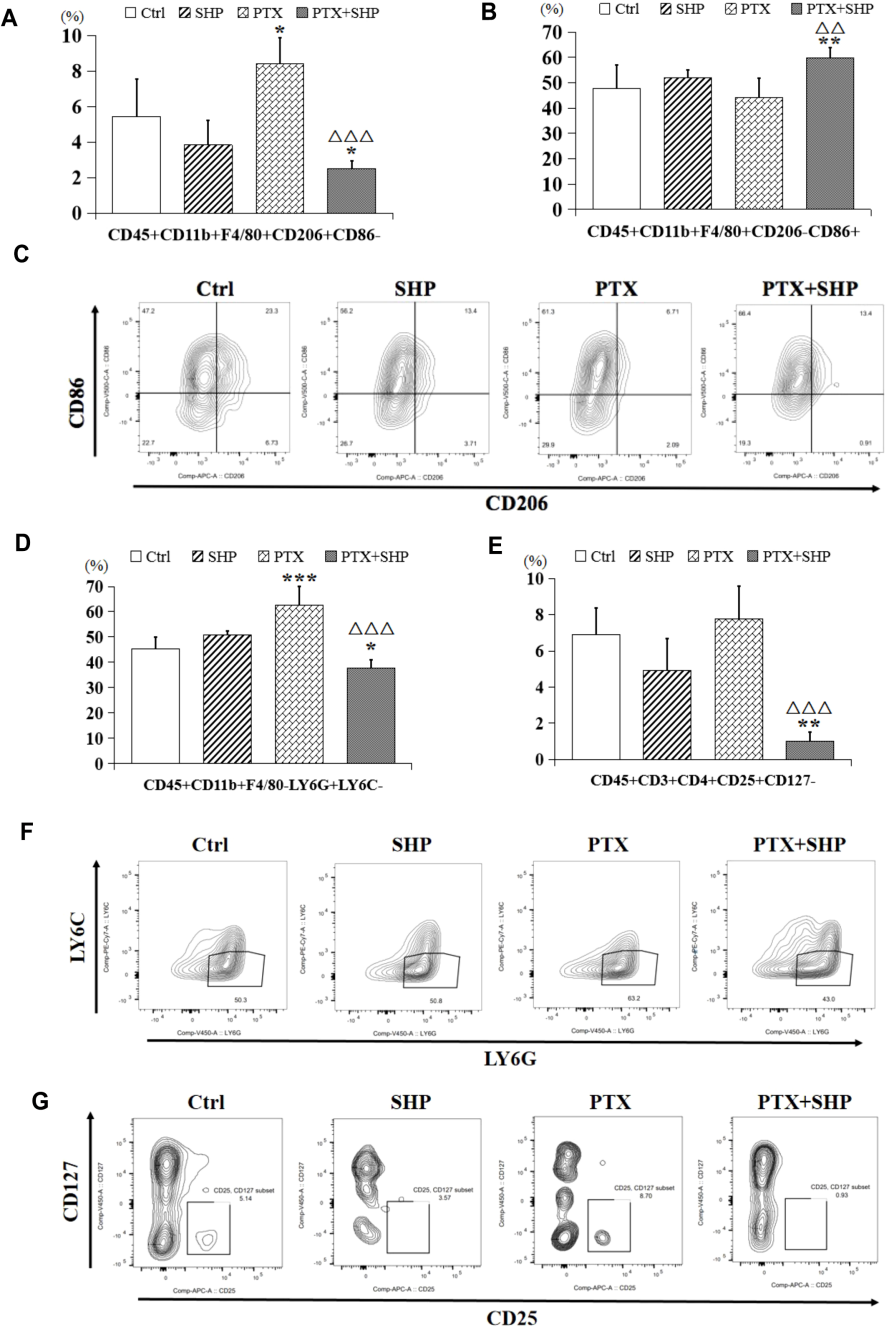
The combination of SHP and PTX suppresses the infiltration of inflammatory cells in solid tumors. **(A)** FACS analysis of CD45+CD11b+F4/80+CD206+CD86- cells (TAMs or M2 macrophages). **(B)** FACS analysis of CD45+CD11b+F4/80+CD206-CD86+ cells (classical macrophages or M1 macrophages). **(C)** Representative FACs data of M1 macrophages and M2 macrophages. **(D)** FACS analysis of CD45+CD11b+F4/80-Ly6G+Ly6C- cells (MDSCs). **(E)** FACS analysis of CD45+CD3+CD4+CD25+CD127- (Tregs). **(F)** Representative FACs data of MDSCs. **(G)** Representative FACs data of Tregs. Data are presented as means ± SD (*P < 0.05, **P < 0.01, and ***P < 0.001 compared to the Ctrl group; △△P < 0.01, and △△P < 0.001 compared to the PTX group).

FACS analysis was performed to assess the impact of SHP combined with PTX on the infiltration of inflammatory cells in solid tumors. The FACS analysis focused on macrophages, MDSCs, and Tregs in the four experimental groups (Ctrl, SHP, PTX, PTX+SHP). The proportion of CD45+CD11b+F4/80+CD206+CD86- cells (M2 macrophages or TAMs) was significantly lower in the PTX+SHP group compared to the PTX group, indicating a reduced infiltration of tumor-promoting macrophages (**Figure 3A**). Conversely, the proportion of CD45+CD11b+F4/80+CD206-CD86+ cells (M1 macrophages, which are antitumorigenic) was significantly higher in the PTX+SHP group, suggesting an enhancement of the antitumor immune response (**Figure 3B**). Representative FACS data further demonstrated these trends (**Figure 3C**).

The proportion of CD45+CD11b+F4/80-Ly6G+Ly6C- cells (MDSCs), which contribute to immunosuppression in the TME, was significantly reduced in the PTX+SHP group compared to the PTX group (**Figure 3D**). This indicates that the combination treatment helps alleviate immunosuppression. The percentage of CD45+CD3+CD4+CD25+CD127- cells (Tregs), known for their immunosuppressive roles, was also significantly reduced in the PTX+SHP group compared to the PTX group (**Figure 3E**). This reduction highlights the immunomodulatory effect of SHP in combination with PTX, promoting a more favorable immune environment. Representative FACS data for MDSCs and Tregs are shown in **Figures 3F, G**, respectively.

These data suggest that the combination of SHP and PTX modulates the TME by decreasing the infiltration of immunosuppressive cells (M2 macrophages, MDSCs, and Tregs) and enhancing the presence of antitumor immune cells (M1 macrophages), both of which contribute to the inhibition of tumor growth.

### 3.4 The combination SHP and PTX enhances T cell and B cell activation in the TME

**Figure 4:**
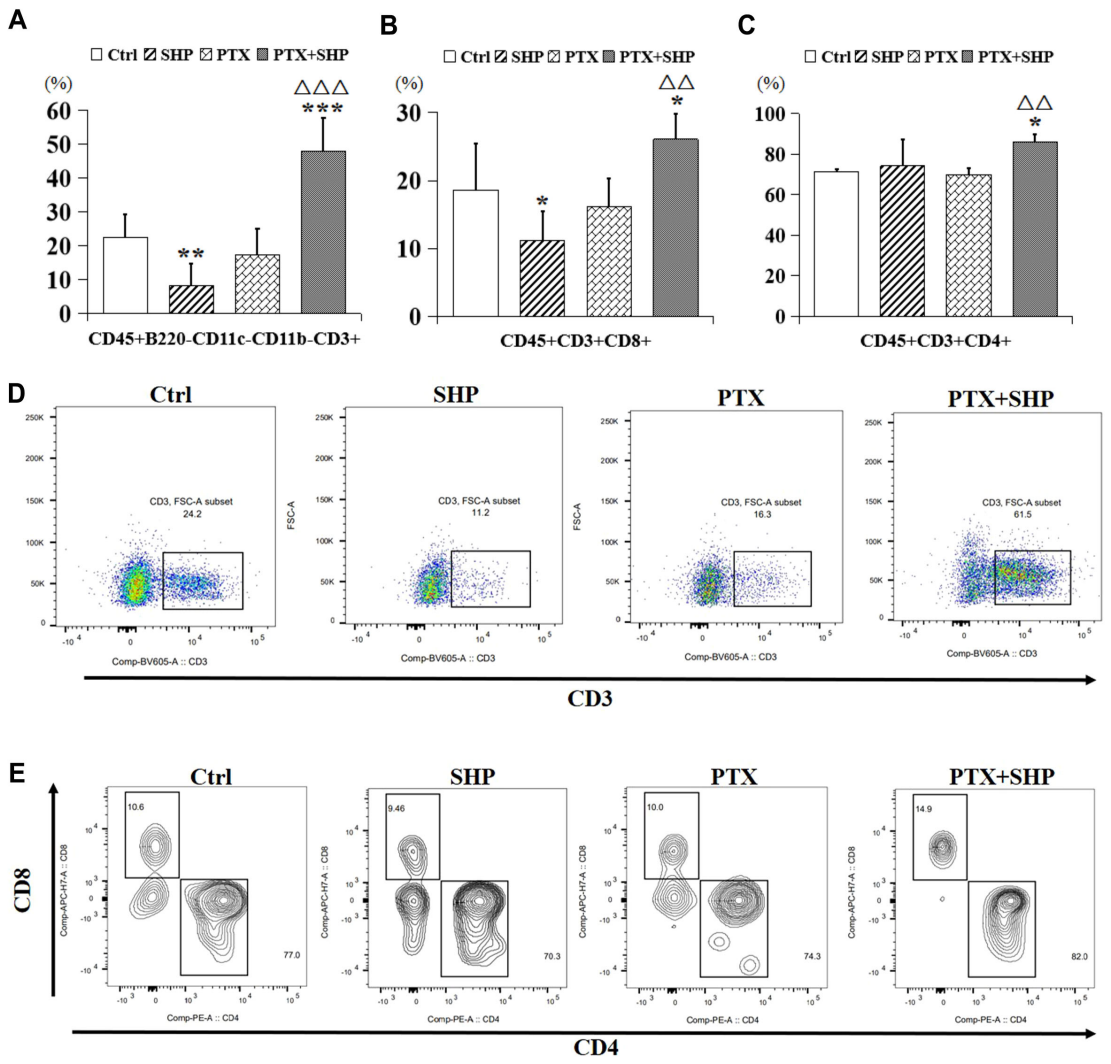
The combination of SHP and PTX increases CD3 and CD4/CD8 positive T cells in the TME. **(A)** FACS analysis of CD45+B220-CD11c-CD11b-CD3+ T cells (CD3+ T cells). **(B)** FACS analysis of CD45+B220-CD11c-CD11b-CD3+CD8+ T cells (CD8+ T cells). **(C)** FACS analysis of CD45+B220-CD11c-CD11b-CD3+ CD4+ T cells (CD4+ T cells). **(D)** Representative FACS data of CD3+ T cells. **(E)** Representative FACS data of CD4+ and CD8+ T cells. Data are presented as means ± SD (*P < 0.05, and ***P < 0.001 compared to the Ctrl group; △△P < 0.01, and △△△P < 0.001 compared to the PTX group).

FACS analysis was performed to evaluate the effects of SHP combined with PTX on T cell populations within the tumor microenvironment (TME). The study focused on CD3+ T cells, CD8+ cytotoxic T cells, CD4+ helper T cells, and B cells across the four experimental groups (Ctrl, SHP, PTX, PTX+SHP). The analysis revealed a significant increase in the proportion of CD45+B220-CD11c-CD11b-CD3+ T cells in the PTX+SHP group compared to the PTX group, indicating enhanced overall T cell infiltration within the TME (**Figure 4A**). Additionally, the proportions of CD45+B220-CD11c-CD11b-CD3+CD8+ T cells (cytotoxic T cells) and CD45+B220-CD11c-CD11b-CD3+CD4+ T cells (helper T cells) were significantly elevated in the PTX+SHP group compared to the PTX group, suggesting improved immune response activation (**Figures 4B, C**). Interestingly, the number of T cells in the SHP group was reduced compared to the Ctrl group, with no significant difference observed between the PTX and Ctrl groups. Representative FACS data confirmed the increase in CD3+ T cells (**Figure 4D**), while further representative data for CD4+ and CD8+ T cells supported these findings (**Figure 4E**).

**Figure 5:**
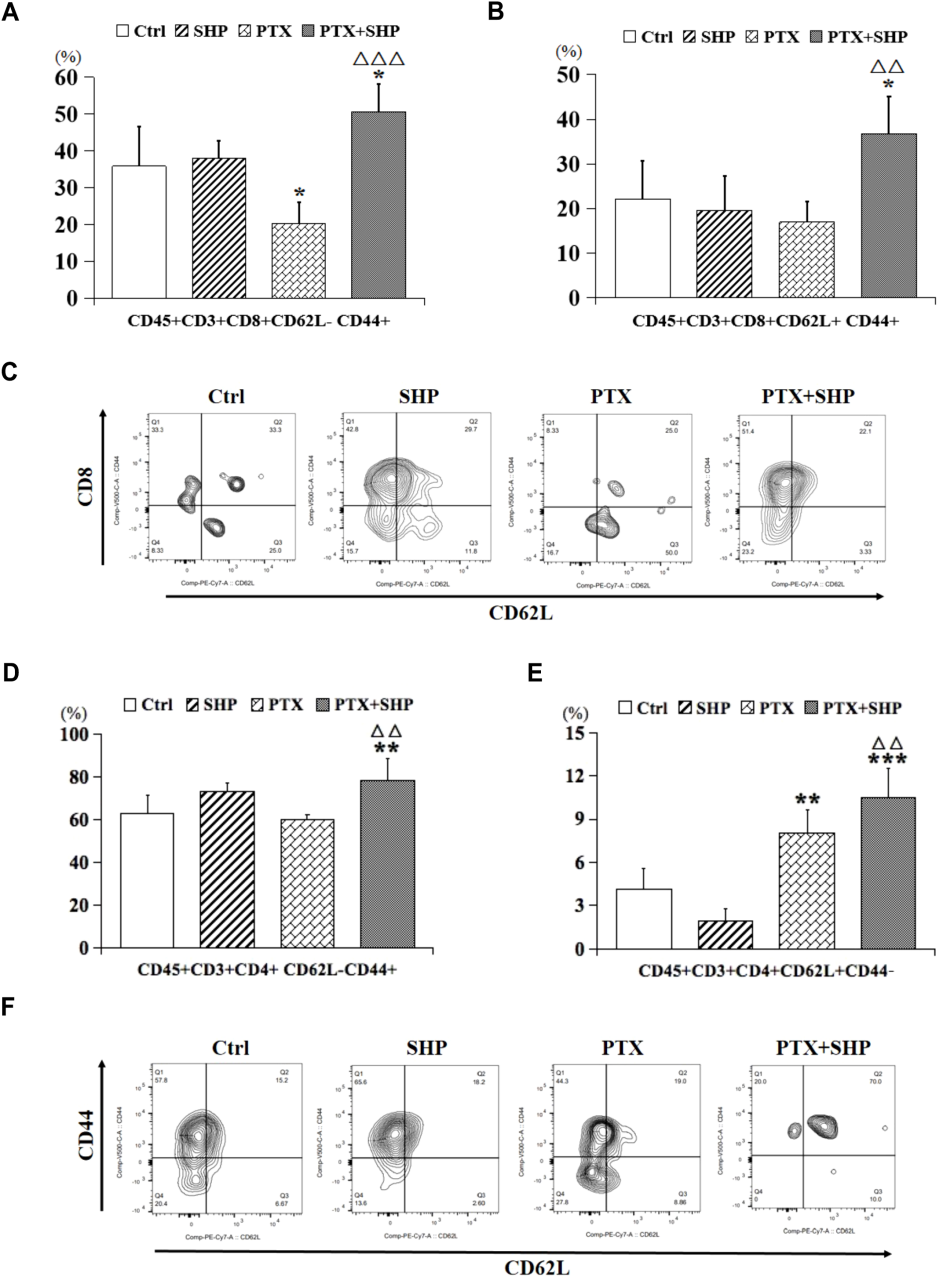
The combination of SHP and PTX increases central memory T cells in the TME. **(A)** FACS analysis of CD45+CD3+CD8+CD62L-CD44+ T cells. **(B)** FACS analysis of CD45+CD3+CD8+ CD62L+CD44+ T cells. **(C)** Representative FACS data of subtypes **(A, B)**. **(D)** FACS analysis of CD45+CD3+CD4+CD62L-CD44+ T cells. **(E)** FACS analysis of CD45+CD3+CD4+CD62L+CD44- T cells. **(F)** Representative FACS data of subtypes **(D, E)**. Data are presented as means ± SD (*P < 0.05, **P < 0.01, and ***P < 0.001 compared to the Ctrl group; △△P < 0.01, and △△△P < 0.001 compared to the PTX group).

**Figure 6:**
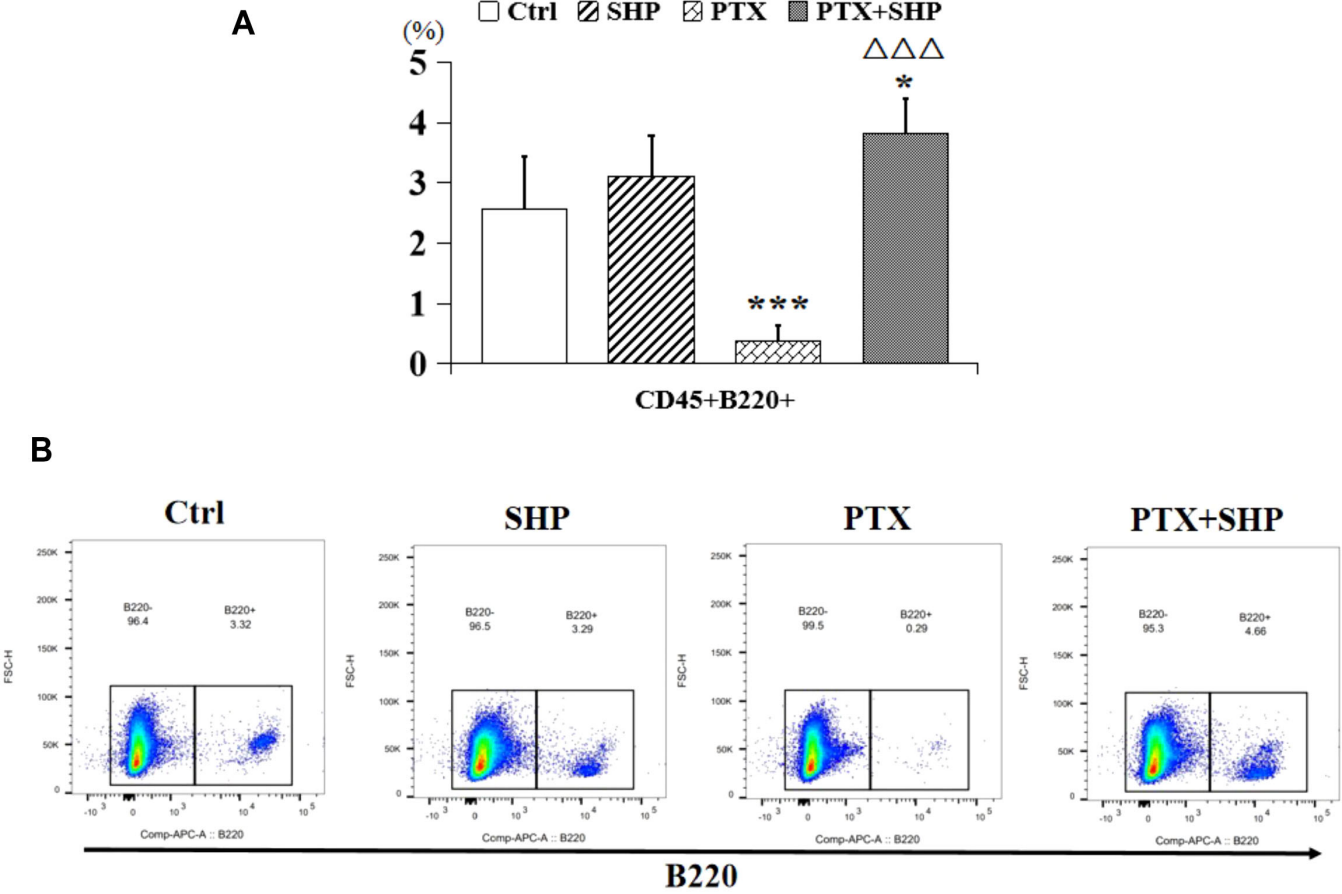
The combination of SHP and PTX promotes the accumulation of B cells in the TME. **(A)** FACS analysis of CD45+B220+ cells (B cells). **(B)** Representative FACS data of B cells. Data are presented as means ± SD (*P < 0.05, ***P < 0.001 compared to the Ctrl group; △△△P < 0.001 compared to the PTX group).

Then, we analyzed the proportion of CD45+CD3+CD8+CD62L+/-CD44+ T cells, which represent central memory T cells, and found that it was higher in the PTX+SHP group compared to the PTX group (**Figures 5A, B**). Representative FACS data of these subtypes are shown in **Figure 5C**. Additionally, CD45+CD3+CD4+CD62L-CD44+ and CD45+CD3+CD4+CD62L+CD44-T cells, which are crucial for long-term immune memory, were significantly increased in the PTX+SHP group (**Figures 5D, E**). Representative FACS data of these subtypes are shown in **Figure 5F**. Finally, FACS analysis of CD45+B220+ cells (B cells) indicated that the PTX+SHP group had a higher proportion of B cells compared to the PTX group (**Figure 6A**). This suggests an enhanced humoral immune response activation in the combination treatment group. Representative FACS data of B cells are shown in **Figure 6B**.

## 4 Discussion

Previous studies have shown that PTX, a neoadjuvant chemotherapy drug, has limited anti-tumor efficacy against advanced 4T1-induced breast cancer in the absence of surgery. In some cases, PTX has been found to promote DM, particularly to the lungs, liver, and bones (18). In agreement with these findings, we observed a significant increase in lung metastases in breast cancer-bearing mice treated with PTX alone. While SHP alone did not delay lung metastasis, the combined use of PTX and SHP led to a marked reduction in lung lesions, as demonstrated by histological analysis. BLI and HE staining also confirmed this reduction. BLI, a highly sensitive technique that can detect as few as 2,500 tumor cells (19), confirmed that there was a significant reduction in tumor volume and metastasis in the PTX+SHP group. Importantly, BLI can accurately measure tumor volume regardless of size or shape and can detect metastases *in vivo* (20).

Our previous research on SHP demonstrated its ability to alleviate PTX-induced constipation by reducing inflammation in the gastrointestinal tract and promoting gastrointestinal motility (16). In the present study, mice treated with PTX alone experienced the most significant weight loss and physical deterioration. Two mice in the PTX group died on Days 9 and 13 following PTX injection, whereas all mice in the other groups survived until the end of the study. This suggests that the combination of PTX and SHP not only prolongs survival but also improves overall health. SHP also significantly mitigated the PTX-induced lung metastasis; however, SHP alone did not exhibit a clear inhibitory effect on metastasis. Thus, the combination of PTX and SHP demonstrates a synergistic effect in inhibiting tumor development.

We also investigated the impact of PTX and SHP on macrophage accumulation in tumors. Tumors are known to polarize TAMs into a protumor (M2) or antitumor (M1) phenotype (21–24). Our study showed that the proportion of M2 macrophages (CD206+ cells), which are associated with tumor growth (25), was significantly lower in the PTX+SHP group compared to the other groups. This suggests that the combination treatment reduces the accumulation of inflammatory macrophages, thereby slowing tumor progression. In contrast, the number of M1 macrophages, which are positively associated with the normalization of the TME (26, 27), increased in the PTX+SHP group.

In terms of immune escape, previous research has demonstrated that MDSCs promote tumor immune evasion and exert a strong immunosuppressive activity in the TME (28–30). MDSCs are categorized into two populations: granulocytic MDSCs (G-MDSCs, CD11b+Ly6G+Ly6C-) and monocytic MDSCs (M-MDSCs, CD11b+Ly6G−Ly6C+) (31, 32). The G-MDSC subtype has been implicated in tumor promotion (28, 33), and MDSCs also suppress natural killer (NK) cell-mediated cytotoxicity, thereby affecting tumor immunosurveillance. Our study revealed a significant reduction in CD11b+Ly6G+Ly6C- cells in the PTX+SHP group, indicating that the combination therapy inhibits tumor immune escape. Furthermore, chronic inflammation within the TME promotes tumor progression (34–36), and we observed a decrease in Tregs (CD4+CD25+CD127-), which are known to support tumor growth and suppress anti-tumor immune responses (37, 38) in the PTX+SHP group.

Tumor-specific CD8+ T cells play a critical role in suppressing tumor growth (39). Our results showed that the percentages of CD3+ T cells, CD8+ T cells, and CD4+ T cells were significantly increased in the PTX+SHP group compared to the PTX group, while no significant differences were observed between the PTX and SHP groups. This indicates that the combination of SHP and PTX effectively enhances the activation of cytotoxic T cells, thereby contributing to tumor inhibition. Notably, CD62L+ T cells, a key subtype of CD8+ and CD4+ T cells involved in preventing DM (40), were significantly increased in the PTX+SHP group. Additionally, the percentage of B220+ B cells, which are important for the humoral immune response, followed a similar trend, further highlighting the immunomodulatory effects of the combination therapy.

In conclusion, the results of this study suggest that PTX and SHP have a synergistic anti-tumor effect, likely through their combined action on immune pathways within the TME. This includes inhibiting angiogenesis, reducing inflammatory cell infiltration, and promoting T cell viability. Previous research has shown that inducible nitric oxide synthase-producing cells (likely MDSCs) in mice with lung tumors can be targeted by silibinin (41), which suggests that there is a potential link between these cells and enzymes that affect gastrointestinal motility. Given that SHP promotes gastrointestinal motility, we hypothesize that its mechanism may be related to the regulation of nitric oxide synthase, though further studies are needed to verify this hypothesis.

## Data Availability

The original contributions presented in the study are included in the article/**Supplementary Material**. Further inquiries can be directed to the corresponding authors.
