# Supplementary material for: The combination of Shenhuang plaster and paclitaxel inhibits lung metastasis in breast cancer via modulation of the tumor microenvironment
Source: Front Oncol. 2025 Feb 28;15:1531493. doi: 10.3389/fonc.2025.1531493 (PMC11906457; doi:10.3389/fonc.2025.1531493)
Supplement: Supplementary file 1 [file Table1.docx]

Supplementary Material

# Supplementary Tables

**Supplementary Table 1. Fluorescent flow cytometry antibodies**

| Panel | Antibody | Conjugated fluorescence | Panel | Antibody | Conjugated fluorescence |
| --- | --- | --- | --- | --- | --- |
| Mono | CD45 | FITC- | TEMT | CD45 | FITC- |
|  | CD11b | APC-R700- |  | CD8 | APC-H7- |
|  | F4/80 | PE- |  | CD3 | BV605- |
|  | Ly6C | PE-Cy7- |  | CD44 | V500-C- |
|  | Ly6G | V450- |  | CD62L | PE-Cy7- |
|  | CD86 | V500-C- |  | CD25 | APC- |
|  | CD206 | APC- |  | CD127 | V450- |
| Lymph | CD45 | PE-Cy7- | Lymph | CD8 | APC-H7- |
|  | NK11 | V450- |  | B220 | APC- |
|  | CD3 | BV605- |  | CD11c | PE- |
|  | CD4 | FITC- |  | CD11b | APC-R700 |

**Supplementary Table 2. Cell marker combinations for cell type identification**

| Cell type | | Surface subtypes |
| --- | --- | --- |
| Macrophages | M1 (Classic type) | CD45+CD11b+F4/80+CD206-CD86+ |
|  | M2 (TAMs) | CD45+CD11b+F4/80+CD206+CD86- |
| MDSCs | G-MDSCs (CD11b+Gr-1high) | CD45+CD11b+F4/80-Ly6G+Ly6C- |
|  |  |  |
|  | M-MDSCs (CD11b+Gr-1int) | CD45+CD11b+F4/80-Ly6G−Ly6C+ |
| B cells | | CD45+B220+ |
| CD3+ T cells | | CD45+B220-CD11c-CD11b-CD3+ |
| CD4+CD8+ T cells | | CD45+B220-CD11c-CD11b-CD3+CD8+CD4+ |
| Cytotoxic T lymphocytes | CD4+ T cells | CD45+CD3+CD4+CD62L+ CD44- |
|  | CD8+ T cells | CD45+CD3+CD8+CD62L+CD44- |
| Tregs | | CD45+CD3+CD4+CD25+CD127- |
